# A Series of Polymer-Supported Polyoxometalates as Heterogeneous Photocatalysts for Degradation of Organic Dye

**DOI:** 10.3390/molecules28093968

**Published:** 2023-05-08

**Authors:** Fan Yang, Xiaojiao He, Tingting Xin, Huizhen Yang, Lijie Bai, Lihua Gao, Yibo Wang

**Affiliations:** Department of Chemistry, Beijing Technology and Business University, Beijing 100048, Chinagaolh@th.btbu.edu.cn (L.G.)

**Keywords:** polymer, polyoxometalate, photocatalyst, degradation, methyl red

## Abstract

Photocatalytic degradation technology has developed rapidly in the treatment of organic pollutants due to its high efficiency, mild reaction conditions and easy control. In this paper, a series of heterogeneous photocatalysts, BWZ-en-R (BWZ = [BW_11_Z(H_2_O)O_39_]^7−^, Z = Zn, Cd, Mn, en = ethylenediamine, R = Merrifield resin), were prepared by using ethanediamine as a linker to immobilize Keggin-type transition elements substituting tungstoborates on Merrifield resin and characterized by Fourier transform infrared spectroscopy, X-ray powder diffraction, scanning electron microscopy and energy-dispersive X-ray spectroscopy. The photocatalytic properties of BWZ-en-R (Z = Zn, Cd, Mn) for the degradation of methyl red (MR) were investigated. The results show that the BWZ-en-R (Z = Zn, Cd, Mn) photocatalysts exhibited high photodegradation ability for MR under the irradiation of ultraviolet light, and were easily separated from the reaction media. The maximum degradation rate (%) of MR (40 mL, 25 μM, pH = 2) reached 96.4% for the BWMn-en-R photocatalyst (40 mg) after being irradiated for 30 min, making this a promising photocatalyst candidate for dye degradation. Moreover, the influences of some factors, such as the Z-substituted elements in the BWZ, the BWZ-en-R dosage and the MR initial concentration, on the photocatalytic degradation rate of MR were also examined.

## 1. Introduction 

Along with the development of the economy and society, the utilization rate and demand for water resources are increasing, and therewith also the discharge of sewage, which makes biodiversity and human health problems of major concern [1,2,3]. At present, among water pollutants, organic pollutants are extremely polluting, with the characteristics of wide distribution, multiple types and complex components, such as some dye pollutants [1,4], antibiotics [5,6], phenols [7,8] and other toxic substances. Among them, dye wastewater has the characteristics of high chromaticity, high content, complicated components and biological toxicity, as well as difficulty in biochemical degradation [9]. It is developing towards resistance to photodegradation and oxidation. This further increases the difficulty of treating dye wastewater; thus, their removal from industrial effluents is indispensable. At present, the methods to treat organic dye wastewater include adsorption [10], membrane separation [11], chemical methods [12] and biological methods [13]. In recent years, the photocatalysis method has been increasingly adopted in the degradation and destruction of organic pollutants due to its high efficiency, simplicity and easy control [14,15,16,17,18,19,20,21]. It is a degradation method that utilizes the strongly oxidizing free radicals generated by the catalyst under the action of light to decompose organic pollutants into inorganic small molecules, and thus photocatalysts are certainly key materials.

Polyoxometalates (POMs), containing discrete anionic metal–oxygen clusters, are mainly based on molybdenum and tungsten. Their cage structure, resembling that of zeolite, offers an opportunity for some smaller polar molecules to enter the bulk phase or react on the surfaces of POMs. Thus, POMs possess higher catalytic activity and selectivity. On the other hand, they have promising prospects in catalysis [22,23,24,25] because of their intriguing structural diversity, thermal stability, redox properties, photoactivity, low toxicity and cost. The photocatalysis technique using POMs provides an environmentally friendly method for the degradation of organic pollutants, especially some azo dyes [25,26]. However, the reaction involved in the photocatalytic degradation of organic pollutants occurs in aqueous media. In spite of the many advantages, most POMs used as photocatalysts are soluble in water, so they are difficult to separate and recycle from the reaction system at the end of the reaction. Moreover, the application of POMs in catalysis is limited due to the low surface area (<10 m^2^/g) [27], and the size of the catalyst’s surface area also has a significant effect on the reaction activity. To circumvent the aforementioned problems, many efforts have been devoted to preparing heterogeneous POM-based photocatalysts, such as combination with large counter ions [28], impregnation on SiO_2_ or TiO_2_ [18] and immobilization onto amine-functionalized mesoporous MCM-41 [29] or polymer matrixes [30] and so on. Resins have the functions of exchange, selection and adsorption; thus, they are widely used as carriers in the field of water treatment. Hua et al. [31] prepared a PW_11_Mn/D301R photocatalyst using D301R as a carrier, in which D301R was a macroporous weak basic exchange resin, and reported a comparative study of the photocatalytic activity of Keggin-type PW_11_Mn and PW_11_Mn/D301R. The results showed that the photocatalyst PW_11_Mn/D301R was stable and its photocatalytic degradation rate for RhB was better than that of PW_11_Mn. Lei et al. [32] synthesized PW_12_ immobilized on an anionic exchange resin D201; PW_12_/D201 as a heterogeneous catalyst had excellent stability and reusability. A total organic carbon removal rate of ca. 22% was achieved. Liu et al. [33] also selected resin as the catalytic carrier to immobilize a 12-tungstophosphoric acid (TPA) catalyst in order to study the oxidative desulfurization process. They compared the desulfurization efficiency based on different resins and found that TPA/IRA900C showed the best desulfurization performance. After three cycles of use, the conversion efficiency of dibenzothiophene (DBT) decreased from 95.8% to 87.9%, only 7.9%. It could also be seen that the photocatalytic activity and reusability of the materials can be effectively improved by loading POMs onto resins. However, stable and efficient POM-based photocatalysts involving resins for the degradation of organic pollutants are still limited. In particular, a few studies on the photocatalytic behavior of catalysts based on POMs containing boron were carried out [34,35]. Thus, it is very worthwhile to carry out research on tungstoborate catalysts in an attempt to establish a structure–photocatalytic performance relationship.

**Figure 1:**
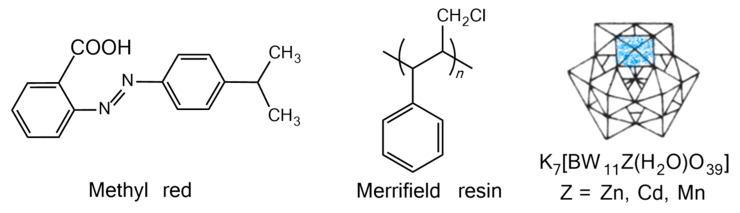
The structures of methyl red (MR), Merrifield resin (R) and K_7_[BW_11_Z(H_2_O)O_39_] (Z = Zn^2+^, Cd^2+^, Mn^2+^, BWZ).

We investigated the photocatalytic activity of some POMs for the degradation of organic dyes, such as K_7_[BW_11_O_39_Z(H_2_O)] (Z = Zn, Cd) [35], H_7_[P_2_Mo_17_VO_62_] [36] and 1,3-bis(carboxymethyl)imidazolium phosphotungstate [37], and observed that the structures and compositions of the POMs had significant effects on their photocatalytic properties. Herein, we prepared a series of heterogeneous photocatalysts, BWZ-en-R, by using ethanediamine (en) as a linker to immobilize Keggin-type transition elements substituting tungstoborates [BW_11_Z(H_2_O)O_39_]^7−^ (denoted as BWZ, Z = Zn, Cd, Mn) on Merrifield resin (denoted as R). Methyl red (MR), as one of the azo dyes with carcinogenicity, is a neutral molecule, and it is less prone to charge interactions with catalysts. Thus, MR was selected as the target organic pollutant to investigate the photocatalytic properties of BWZ-en-R under UV light irradiation. Figure 1 provides the structures of the BWZ, R and MR that we used in this work.

## 2. Results and Discussion

### 2.1. Preparation and Characterization of Photocatalysts

**Figure 2:**
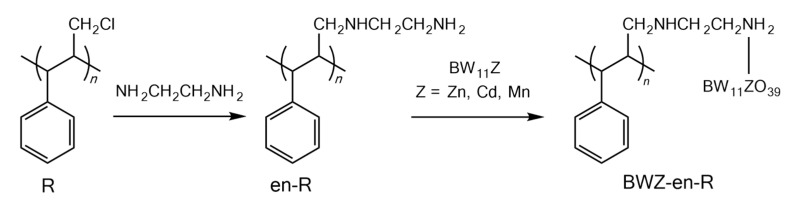
Synthetic route of BWZ-en-R (Z = Zn, Cd, Mn).

In this work, Merrifield resin (R) as a solid support for BWZ (Z = Zn, Cd, Mn) was constructed of polystyrene with chloromethyl groups at the side of the main chain. Since chloromethyl groups in the R molecule can react with NH_2_- groups in the ethylenediamine (en) to form en-modified Merrifield resin (en-R) with NH_2_- groups, the BWZ (Z = Zn, Cd, Mn) was loaded onto the R by replacing coordinated water in the BWZ molecule with NH_2_- groups in the en-R (Figure 2). The maximum loading amount of BWZ on the R was ca. 0.1 g/g resin.

**Figure 3:**
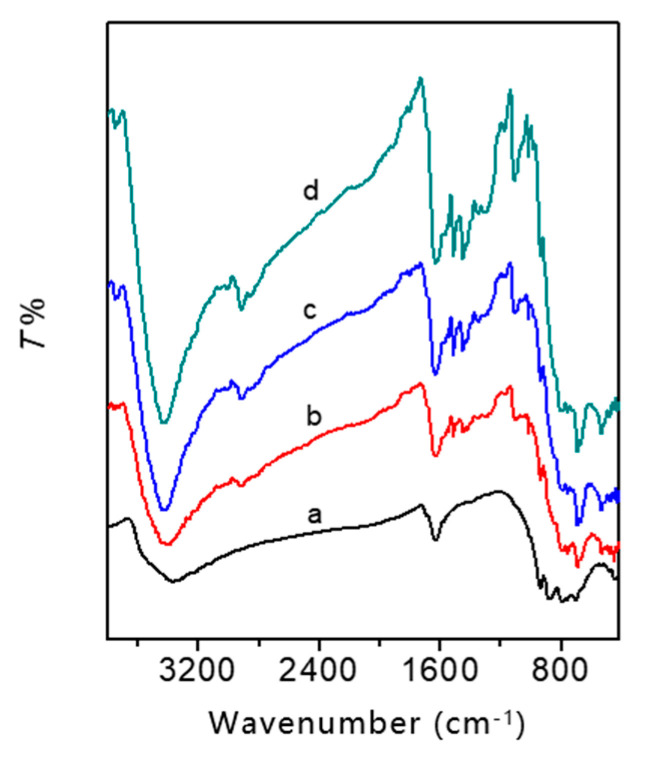
IR spectra of BWZn (a), BWZ-en-R (Z = Zn (b), Cd (c), Mn (d)).

Fourier infrared (FT-IR) spectroscopy is very useful in the characterization of POMs; thus, it was employed to gather structural information about BWZ in BWZ-en-R. Figure 3 shows the FT-IR spectra of BWZ-en-R (M = Zn, Cd, Mn) (traces b, c and d) together with the spectrum of the parent BWZn (trace a) for comparison. For the parent BWZ, the characteristic peaks are mainly found at 938~955, 883~885, 800~811, 735~787 cm^−1^, which are assigned to the asymmetric stretch vibrations of W = O_t_ (O_t_-terminal), B-O_i_ (O_i_-internal), W-O_e_-W (O_e_-edge-sharing) and W-O_c_-W (O_c_-corner-sharing), respectively [35,38]. Compared to the parent BWZ, the BWZ-en-R (M = Zn, Cd, Mn) in the range of 1100-700 cm^−1^ also exhibited four characteristic peaks of Keggin anions at around 938, 850, 802 and 695 cm^−1^, suggesting that the Keggin-type BWZ (M = Zn, Cd, Mn) anions were intact in the BWZ-en-R (M = Zn, Cd, Mn) photocatalysts [38]. Moreover, the peaks between 1700 and 1400 cm^−1^ contributed to the skeleton vibration of the benzene ring in the Merrifield resin R; the peaks at 3400 cm^−1^ were designated as stretching vibrations of N-H in the ethylenediamine component; the peaks at ~2900 and ~2800 cm^−1^ were designated as stretching vibrations of -CH_2_ in the R and en components. The above results demonstrate the coexistence of BWZ, R and en components in BWZ-en-R (M = Zn, Cd, Mn). 

**Figure 4:**
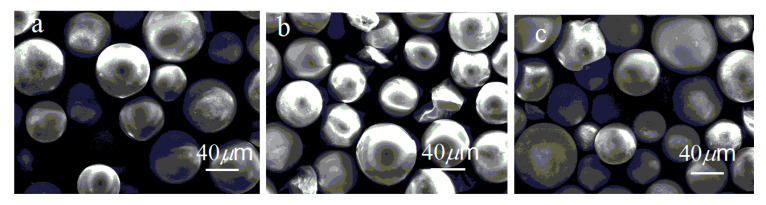
SEM images of BWZn-en-R (**a**), BWCd-en-R (**b**) and BWMn-en-R (**c**).

To obtain the morphology and component information of the photocatalysts, the morphology of BWZ-en-R (M = Zn, Cd, Mn) was characterized using scanning electron microscopy (SEM), as shown in Figure 4. It can be seen that BWZ-en-R had a spherical shape with quite a smooth surface, and the spheres were nearly monodisperse in size, with ca. 30–60 μm diameter, which is characteristic of Merrifield resin R. In order to confirm the existence of the BWZ component in BWZ-en-R, energy-dispersive X-ray spectroscopy (EDS) measurement was carried out (see Figure S1). The EDS of BWZ-en-R (M = Zn, Cd, Mn) exhibited diffraction peaks of element W at 1.82, 2.15 and 8.38 keV, indicating that BWZ was successfully immobilized on the Merrifield resin. 

The X-ray diffraction (XRD) patterns of three BWZ-en-R (Z = Zn, Cd, Mn) are shown in Figure S2, which shows that the patterns were very similar to each other. This indicates that BWZ-en-R has no characteristic diffraction peak because of the Merrifield resin’s amorphous structure. Moreover, no diffraction peaks related to the Keggin unit of BWZ were observed in the XRD pattern of BWZ-en-R, implying the homogeneous dispersion of the Keggin unit BWZ in the Merrifield resin, without any aggregation.

### 2.2. Photocatalytic Activity of BWZ-en-R

**Figure 5:**
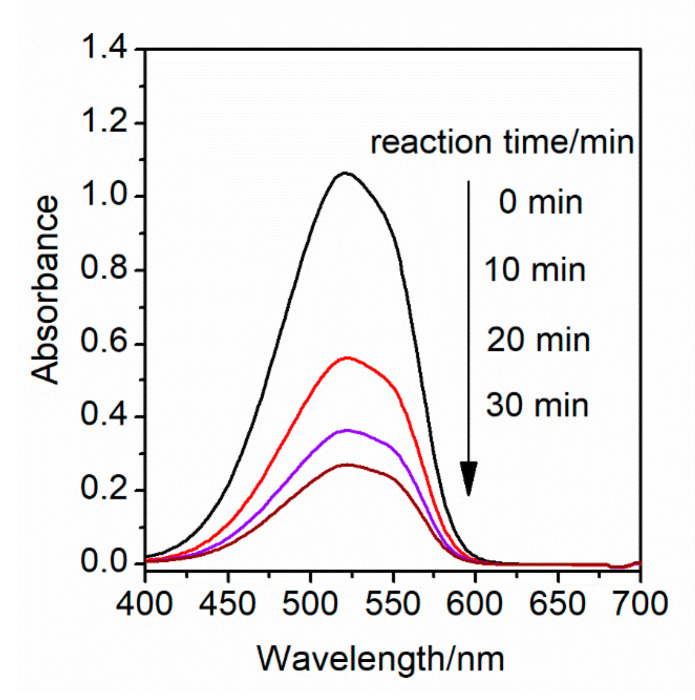
Visible absorption spectra of the MR solution (25 μM, pH = 2) under different irradiation times in the presence of BWZn-en-R (40 mg).

**Figure 6:**
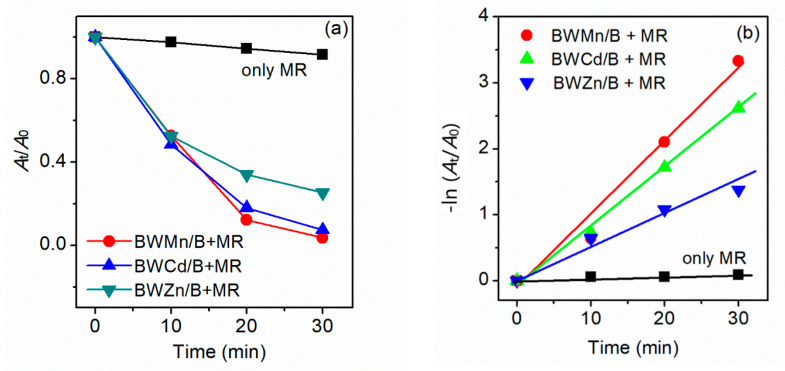
(**a**) Photodegradation efficiency of MR versus irradiation time under ultraviolet irradiation in the presence of BWZ/R (Z = Zn, Cd, Mn) and (**b**) kinetic curves for the photodegradation of MR.

**Figure 7:**
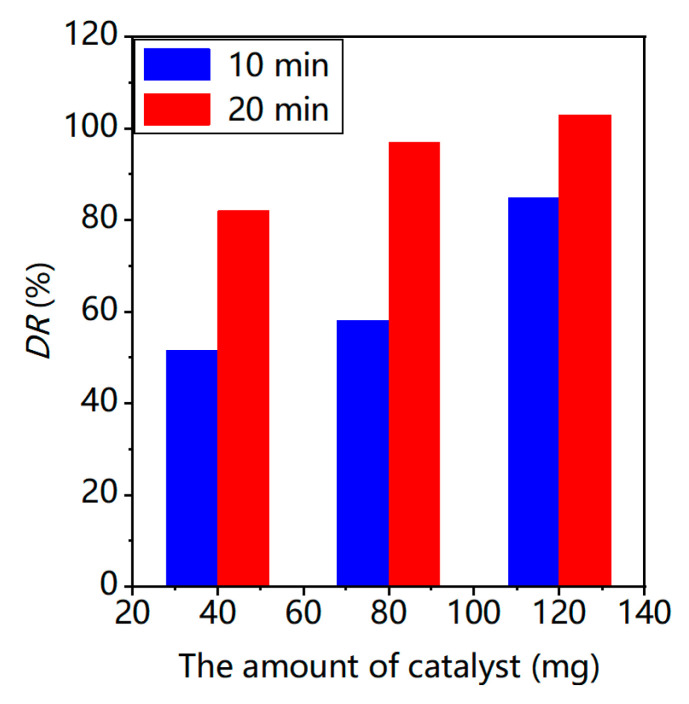
The degradation rate of 25 μM MR (pH = 2, 40 mL) versus the BWCd-en-R amount after 10 and 20 min ultraviolet light irradiation.

To explore the photocatalytic activity of BWZ-en-R (Z = Zn, Cd, Mn) for the degradation of organic pollutants, MR was selected as a model dye pollutant. The degradation process of the MR was monitored spectrophotometrically by measuring the absorption spectra of the MR aqueous solution. The concentration change of the MR in the degradation process was estimated based on the characteristic absorption peak of the MR at 520 nm. The degradation reaction of the MR solution (40 mL, 25 μM, pH = 2) was not observed in the presence of BWZ-en-R (40 mg) in the dark. Even under sunlight irradiation, the MR was still difficult to degrade in the presence of BWZ-en-R (40 mg). Additionally, the degradation rate (DR) of the MR was only 8.5% in the absence of BWZ-en-R under ultraviolet irradiation for 20 min. Therefore, the BWZ-en-R catalysts and ultraviolet (UV) light are indispensable for an efficient MR degradation process. In this study, the photocatalytic activity of BWZ-en-R (Z = Zn, Cd, Mn) was evaluated via the degradation of the MR aqueous solution under ultraviolet irradiation. As a representative example, Figure 5 shows the visible absorption spectra of the MR solution (40 mL, 25 μM, pH = 2) degraded by the BWZn-en-R (40 mg) photocatalyst under UV irradiation at different irradiation times. It can be seen that the characteristic absorption peak of the MR solution at 520 nm successively decreased with the increasing time of irradiation, indicating that the MR degraded easily in the presence of BWZn-en-R under ultraviolet irradiation. Figure 6a presents the curves of A_t_/A_0_ of the MR solution (40 mL, 25 μM, pH = 2) with the irradiation time using the BWZn-en-R, BWCd-en-R and BWMn-en-R photocatalysts, respectively. As shown in Figure 6a, after being irradiated for 30 min, the DR (%) of the MR using the BWZn-en-R, BWCd-en-R and BWMn-en-R photocatalysts reached 75.0%, 92.7% and 96.4%, respectively. Compared with the self-degradation of MR without the photocatalyst, the DR (%) of the MR for BWZn-en-R, BWCd-en-R and BWMn-en-R increased to 66.5%, 84.2% and 87.9%, indicating that the three BWZ-en-R (Z = Zn, Cd, Mn) catalysts had good photocatalytic activity for the degradation of MR. In addition, the BWZ-en-R (Z = Zn, Cd, Mn) used for the degradation of MR also exhibited significantly better photocatalytic activity compared with 1,3-bis(carboxymethyl)imidazolium phosphotungstate, which was previously reported [37], indicating that the composition of the POM had a remarkable influence on its photocatalytic activity. The DR (%) of the MR was greater than 90% for the BWCd-en-R and BWMn-en-R photocatalysts, which was 1.24 and 1.29 times that of BWZn-en-R, demonstrating that BWCd-en-R and BWMn-en-R are more efficient photocatalysts for the degradation of MR than BWZn-en-R. Obviously, the different photocatalytic activity of BWZn-en-R, BWCd-en-R and BWMn-en-R is caused by the different substituted element Z in the BWZ component. In addition, we observed that the photocatalytic activity of BWZ-en-R was also influenced by the dosage of the photocatalyst and the initial concentration of the MR solution. Figure 7 gives the rate of MR photodegradation as a function of the BWCd-en-R dosage after 10 and 20 min of UV-light irradiation. It can be seen that the rate of the MR photodegradation increased with the increase in the BWCd-en-R dosage from 40 to 120 mg. This is due to the fact that the active sites of the photocatalyst increased with the increasing photocatalyst amount, resulting in the increased catalytic activity of the photocatalyst. When the BWCd-en-R dosage was increased to 120 mg, 40 mL of 25 μM MR solution was completely degraded after 20 min. 

**Figure 8:**
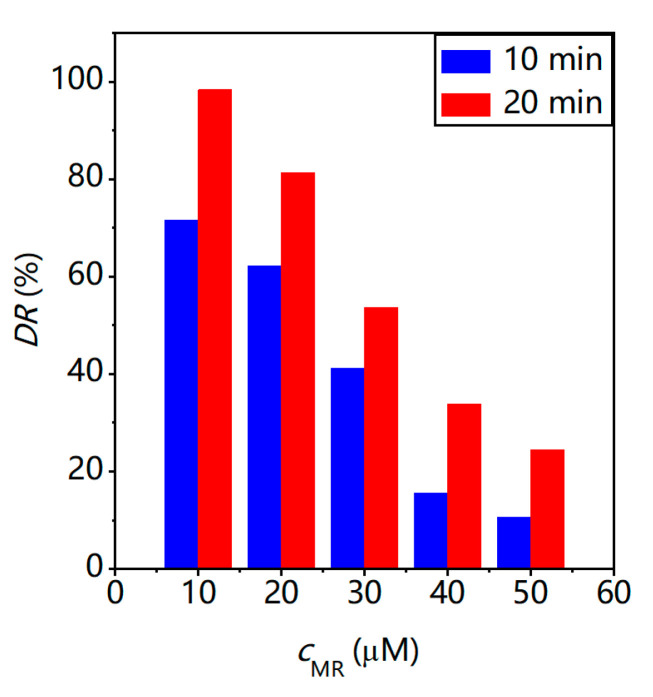
The degradation rate of 25 μM MR (pH = 2, 40 mL) with different initial concentrations of MR.

The effect of the MR concentration on the rate of photocatalytic degradation was studied by varying the initial MR concentration at a fixed amount of catalyst. Figure 8 shows the relationship among the rate of photocatalytic degradation and different initial concentrations of the MR solution (40 mL, 10 to 50 μM) after 10 and 20 min UV-light irradiation when BWZn-en-R (40 mg) was used as a photocatalyst. It was found that the DR (%) of the MR was greatly influenced by the initial concentration of the MR solution. When the initial concentration of the MR solution increased from 10 μM to 50 μM, the DR (%) of the MR decreased from 99% to 23% in 20 min. Obviously, it is easier to degrade a low-concentration MR solution using a photocatalyst because when the initial concentration of the MR solution is lower, the active sites available on the catalyst surface are more than required. At a high concentration of the MR solution, the surface of the photocatalyst may be covered by multiple MR layers, disturbing the electron-transfer process and photocatalytic cycle [20], resulting in the rate of MR photocatalytic degradation decreasing. Similar results were obtained by other researchers [39,40]. 

### 2.3. Kinetic Study of Dye Degradation in the Presence of BWZ-en-R

The apparent rate constant (*k*_app_, min^−1^) of a photocatalytic reaction can indicate the catalytic effectiveness of a photocatalyst under the reaction conditions employed [31,38]. To further evaluate the photocatalytic activity of the BWZ-en-R (Z = Zn, Cd, Mn) photocatalysts, the photocatalytic degradation reaction followed the pseudo first-order kinetics equation when the concentration of dye was low [34,41]. The apparent rate constants (*k*_app_, min^−1^) were calculated by the following formula:

(1) $$-\ln\left(\frac{C_{t}}{C_{0}}\right)=-\ln\left(\frac{A_{t}}{A_{0}}\right)=k_{\mathrm{app}}t$$

where *A*_0_ and *A*_t_ are the absorbance of the MR solution at 520 nm at an irradiation time of 0 and t min, respectively. Figure 6b shows the linear relationship of −ln(*A*_t_/*A*_0_) versus the irradiation time for the degradation reaction of MR in the presence of BWZ-en-R (Z = Zn, Cd, Mn), and in the absence of a photocatalyst. The *k*_app_ values can be calculated from the slope of the linear relationship in Figure 6b. The excellent fitness indicates that these photocatalytic reactions follow the first-order reaction kinetics. The *k*_app_ values of the MR degradation reactions for the BWZn-en-R, BWCd-en-R and BWMn-en-R photocatalysts were 0.046, 0.088 and 0.115 min^−1^, respectively. However, the *k*_app_ value in the absence of a photocatalyst was only 0.0026 min^−1^. The results are shown in Table 1 and indicate that BWZ-en-R (Z = Zn, Cd, Mn) exhibits remarkable photocatalytic performance for the degradation of MR. Among different POMs, the photocatalytic activity for the degradation of MR followed the sequence of BWMn-en-R > BWCd-en-R > BWZn-en-R, in which BWMn-en-R is the most efficient photocatalyst. Colak et al. reported the photocatalytic properties of a boron-centered Keggin-type POM, K_16_[Ni(H_2_O)_6_]_2_[BW_12_O_40_]_4_·48H_2_O, for the degradation of MR [34]. K_16_[Ni(H_2_O)_6_]_2_[BW_12_O_40_]_4_·48H_2_O is a promising catalyst candidate for dye degradation, and its value of *k*_app_ for the degradation of MR is 0.072 min^−1^, while the values of *k*_app_ for the degradation of MR with BWMn-en-R and BWCd-en-R in this paper were 1.6 and 1.2 times this value. The obtained results show that BWMn-en-R and BWCd-en-R possess remarkable catalytic activity for the degradation of MR.

### 2.4. Stability of BWZ-en-R

The stability of catalysts is of great significance in their practical application. Therefore, the stability performance of the three polymer-supported photocatalysts after the catalytic degradation of MR was studied. The XRD patterns of BWZ-en-R (Z = Zn, Cd, Mn) before and after the photocatalytic degradation reaction are shown in Figure S3, respectively. Through comparison, it was found that the BWZ-en-R structure did not obviously change in its peak position and shape after the photocatalytic degradation process, and only the crystallinity of BWZ-en-R photocatalysts had some minor variations. The experimental results indicate that the BWZ-en-R (Z = Zn, Cd, Mn) photocatalysts possess good stability and reusability for the degradation of MR and therefore have great potential in the treatment of dye wastewater.

## 3. Experimental Section

### 3.1. Materials

Merrifield resin (R), ethylenediamine (en), 1,4-dioxane and methyl red (MR) were purchased from the Shanghai Yuan Ye Limited Company of Biology Science and Technology and Beijing Chemical Works, respectively, and used as received, without further purification. All chemicals were of analytical reagent grade.

### 3.2. Preparation of BWZ-en-R Photocatalysts

The preparation route of BWZ-en-R is shown in Figure 2. The preparation process was composed of three steps, and BWZ (Z = Zn, Cd, Mn) was synthesized according to the method reported by our group [35].

First, 4.00 g of Merrifield resin was dispersed in 20 mL of dioxane for 36 h. Then, 10 mL of ethylenediamine was added to the above-mentioned solution. The mixture was heated to 60–70 °C and stirred for 10 h, cooled to room temperature, and then filtered and washed with deionized water several times until the washing solution was neutral; it was subsequently dried in a vacuum at 60 °C. Ethylenediamine-modified Merrifield resin (abbreviated as en-R) was obtained for further usage.

BWZ (0.125 g) and the as-prepared en-R (1.25 g) were added to 25 mL of deionized water. After 24 h of stirring at room temperature, the product was collected and washed several times with deionized water, and then dried at room temperature. The prepared photocatalysts were labeled BWZ-en-R (Z = Zn, Cd, Mn), in which BWZn-en-R and BWCd-en-R were white, and BWMn-en-R was light brown. The loading capacity of BWZ in the Merrifield resin was approximately 0.1 g/g.

### 3.3. Characterization of Photocatalysts

The FT-IR spectra were measured with a Thermo Nicolet, Avatar 370 spectrophotometer. Scanning electron microscopy (SEM) images and energy-dispersive X-ray (EDX) spectroscopy profiles were obtained on a TESCAN VEGA-II. X-ray power diffraction (XRD) patterns were recorded by a Shimadzu XRD-6000 diffractometer using Cu Kα irradiation (λ = 0.15418 nm), and the applied current and voltage were 30 mA and 30 kV, respectively. 

### 3.4. Photocatalytic Experiments

The photocatalytic activity of BWZ-en-R (Z = Zn, Cd, Mn) under ultraviolet (UV) irradiation from a 500 W high-pressure ultraviolet mercury lamp was evaluated via the degradation of an MR aqueous solution. The photocatalytic reaction was conducted in a cylindrical glass vessel with an XPA-I photochemical reactor (Nanjing Xujiang Machine-Electronic Plant). In the typical experiment, BWZ-en-R (40 mg) was added to MR (40 mL, 25 μM, pH = 2) aqueous solution. Before illumination, the mixed suspension was magnetically stirred in the dark for 1 h to achieve adsorption–desorption equilibrium between BWZ-en-R and MR. Subsequently, the solution was irradiated under UV-light irradiation and stirred frequently during the process, without heating. At given time intervals of illumination, approximately 4 mL of the supernatant was taken out, and its visible spectrum was recorded on a UV–vis spectrophotometer (UV-1100). The concentration of dye solution is linearly proportional to the intensity of the characteristic peak when the concentration is not high. In other words, the stronger the intensity of the characteristic peak of the sample, the greater the concentration of the solution. The concentration of the MR solution was indirectly characterized by measuring the absorption spectrum after photocatalytic degradation. The characteristic absorption peak of MR at 520 nm was used to determine the extent of its degradation. The degradation rate (DR) of MR was calculated according to the following formula [42,43]:

(2) $$\mathrm{DR}\left(\%\right)=\frac{\left(A_{0}-A_{t}\right)}{A_{0}}\times 100\%$$

where *A*_0_ and *A*_t_ are the absorbance of the MR solution at 520 nm before degradation and at time t after degradation.

In addition, in order to further investigate the effects of different factors on the photocatalytic degradation of MR, the principle of controlling variables was adopted to study the degradation degree of MR under 500 W mercury lamp irradiation. Comparative experiments were carried out by changing the type of catalyst (BWZn-en-R, BWCd-en-R, BWMn-en-R), the content of catalyst (40 mg, 80 mg, 120 mg) and the initial concentration of MR (10 μM, 20 μM, 30 μM, 40 μM, 50 μM).

After the photocatalytic degradation of MR, the BWZ-en-R photocatalysts in the heterogeneous reaction were filtered out and washed multiple times with deionized water and ethanol. Subsequently, they were dried in a vacuum drying oven at 60 °C for 1 h, and finally stored at room temperature for reuse. Meanwhile, the stability of BWZ-en-R (Z = Zn, Cd, Mn) after degradation was tested using XRD analysis technology.

## 4. Conclusions

A series of new Keggin-type polyoxometalate-resin heterogeneous photocatalysts, based on BWZ-en-R, have been prepared and structurally characterized. The degradation reactions of MR have been investigated using BWZ-en-R as a photocatalyst under various conditions. It was found that the reaction kinetics followed the pseudo-first-order rate law, and the values of *k*_app_ for the degradation reactions of MR in the presence of BWZ-en-R were between 0.064 min^−1^ and 0.115 min^−1^. Moreover, these polymer-supported photocatalysts are environmentally friendly and easy to separate from reactant media, as demonstrated by recycling. BWZ-en-R exhibits remarkable photocatalytic activity for the degradation of MR under UV irradiation, and the dosage of the catalyst directly affects the photocatalytic degradation rate to a certain degree. Increasing the dosage of the catalyst greatly improves the degradation degree of MR. Notably, BWMn-en-R, as a heterogeneous photocatalyst for the degradation of MR, is superior to other POM photocatalysts in many cases. Therefore, BWMn-en-R photocatalysts are expected to be widely used in dye wastewater treatment systems.

## Figures and Tables

**Table 1:** Degradation rates (DR), reaction rate constants (*k*_app_) and correlation coefficients (*R*^2^) of photocatalysts.

Photocatalyst	DR (%)	*k*_app_ (min^−1^)	*R* ^2^
None		2.6 × 10^−3^	0.8
BWZn-en-R	75.0%	4.6 × 10^−2^	0.96
BWCd-en-R	92.7%	8.8 × 10^−2^	1.00
BWMn-en-R	96.4%	11.5 × 10^−2^	0.97

## Data Availability

Not applicable.

## Supplementary Materials

The following supporting information can be downloaded at: https://www.mdpi.com/article/10.3390/molecules28093968/s1. Figure S1. EDS spectra of BWM-en-R (M = Zn, Cd, Mn). Figure S2. XRD patterns of BWZn and BWZn-en-R (a), BWCd and BWCd-en-R (b), BWMn and BWMn-en-R (c). Figure S3. XRD patterns of BWZn-en-R (a), BWCd-en-R (b) and BWMn-en-R (c) before and after photocatalytic degradation of MR.

## Sample Availability

Samples of the compounds BWZ and BWZ-en-R (Z = Zn, Cd, Mn) are available from the authors.
