# Supplementary material for: A Series of Polymer-Supported Polyoxometalates as Heterogeneous Photocatalysts for Degradation of Organic Dye
Source: Molecules. 2023 May 8;28(9):3968. doi: 10.3390/molecules28093968 (PMC10179983; doi:10.3390/molecules28093968)
Supplement: Supplementary file 1 [file molecules-28-03968-s001.zip › molecules-2366891-supplementary.pdf]

A Series of Polymer-Supported Polyoxometalates as Heterogeneous Photocatalysts for Degradation of Organic Dye

Fan Yang, Xiaojiao He, Tingting Xin, Huizhen Yang, Lijie Bai, Lihua Gao and Yibo Wang *

Department of Chemistry, Beijing Technology and Business University, Beijing 100048, China;

*   Correspondence: wangyb@th.btbu.edu.cn

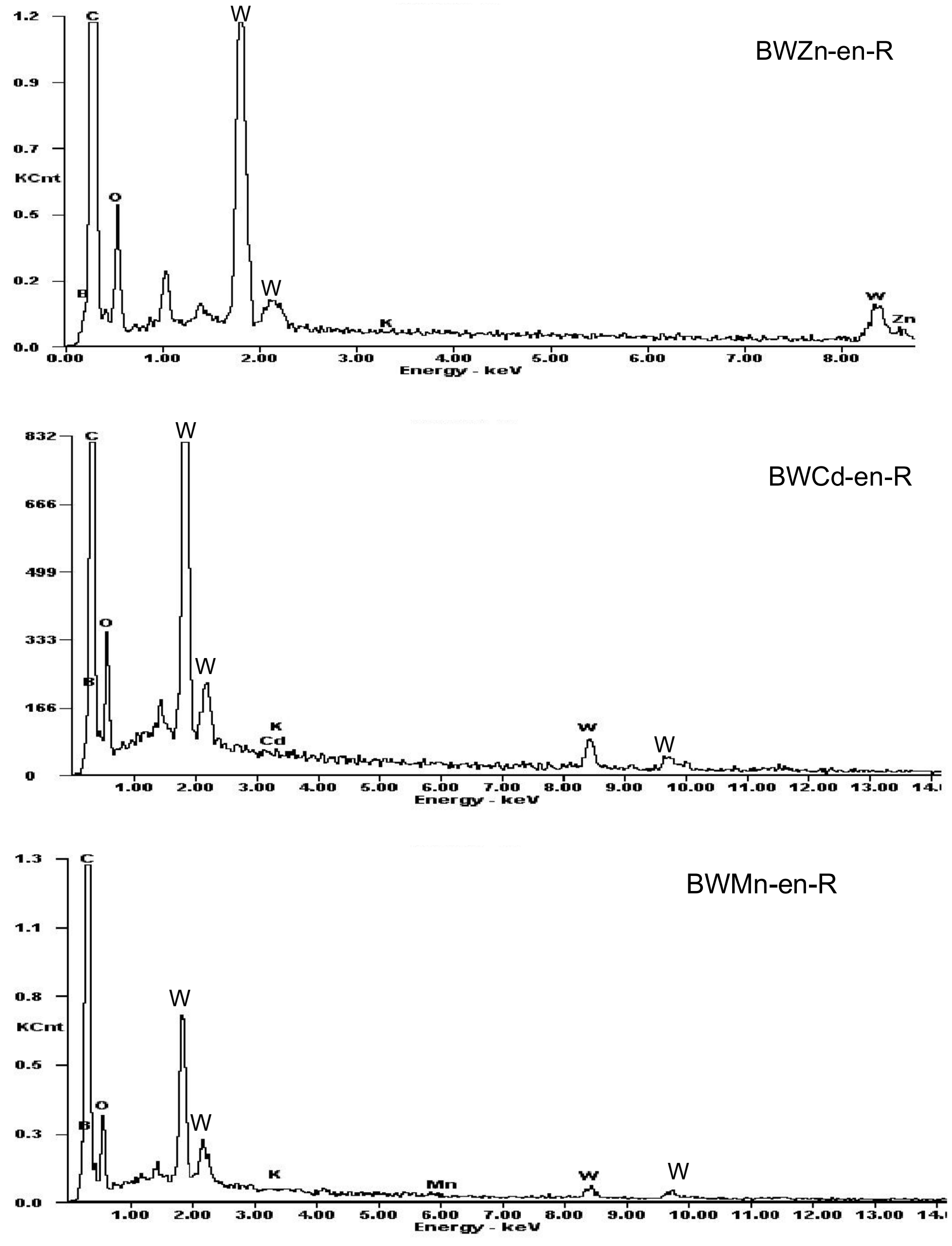

Figure S1. EDS spectra of BWM-en-R (M = Zn, Cd, Mn).

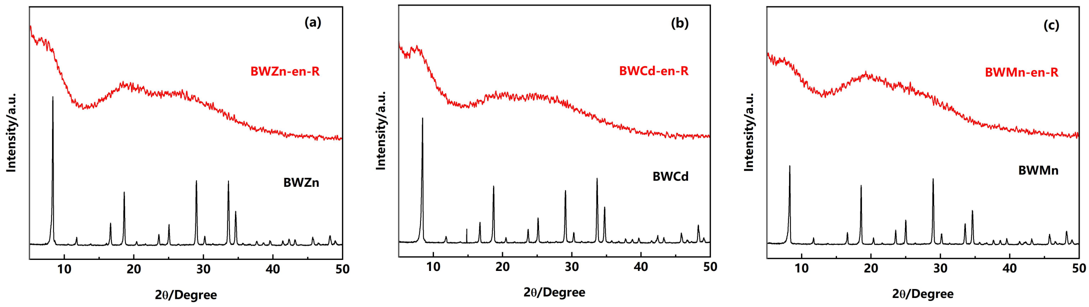

Figure S2. XRD patterns of BWZn and BWZn-en-R (a), BWCd and BWCd-en-R (b), BWMn and BWMn-en-R (c).

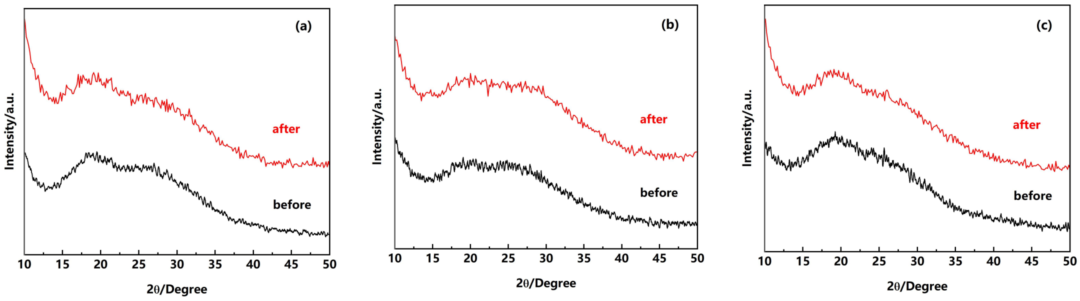

Figure S3. XRD patterns of BWZn-en-R (a), BWCd-en-R (b) and BWMn-en-R (c) before and after photocatalytic degradation of MR.
